# Genomics of Endometriosis: From Genome Wide Association Studies to Exome Sequencing

**DOI:** 10.3390/ijms22147297

**Published:** 2021-07-07

**Authors:** Imane Lalami, Carole Abo, Bruno Borghese, Charles Chapron, Daniel Vaiman

**Affiliations:** Institut Cochin, U1016 INSERM, UMR8104 CNRS and Université de Paris, 24 rue du Faubourg St-Jacques, 75014 Paris, France; imane.lalami@inserm.fr (I.L.); carole.abo@aphp.fr (C.A.); bruno.borghese@aphp.fr (B.B.); charles.chapron@aphp.fr (C.C.)

**Keywords:** endometriosis, genome-wide association studies, exome sequencing, missing heritability, infertility

## Abstract

This review aims at better understanding the genetics of endometriosis. Endometriosis is a frequent feminine disease, affecting up to 10% of women, and characterized by pain and infertility. In the most accepted hypothesis, endometriosis is caused by the implantation of uterine tissue at ectopic abdominal places, originating from retrograde menses. Despite the obvious genetic complexity of the disease, analysis of sibs has allowed heritability estimation of endometriosis at ~50%. From 2010, large Genome Wide Association Studies (GWAS), aimed at identifying the genes and loci underlying this genetic determinism. Some of these loci were confirmed in other populations and replication studies, some new loci were also found through meta-analyses using pooled samples. For two loci on chromosomes 1 (near CCD42) and chromosome 9 (near CDKN2A), functional explanations of the SNP (Single Nucleotide Polymorphism) effects have been more thoroughly studied. While a handful of chromosome regions and genes have clearly been identified and statistically demonstrated as at-risk for the disease, only a small part of the heritability is explained (missing heritability). Some attempts of exome sequencing started to identify additional genes from families or populations, but are still scarce. The solution may reside inside a combined effort: increasing the size of the GWAS designs, better categorize the clinical forms of the disease before analyzing genome-wide polymorphisms, and generalizing exome sequencing ventures. We try here to provide a vision of what we have and what we should obtain to completely elucidate the genetics of this complex disease.

## 1. Introduction

### 1.1. Epidemiology and Phenotypic Description

Endometriosis is a gynecologic disease affecting women of reproductive age. Its precise prevalence is in fact unknown, but classically estimated at around 10%. It is characterized by two major clinical manifestations: pain and infertility. When a patient consults for chronic pelvic pain and infertility, endometriosis is detected in one fourth and one third of the patients, respectively [1].

Pain associated with endometriosis generally increases in intensity at specific moments (during menstruations, dysmenorrhea, dyschezia, lower urinary tract symptoms and/or during intercourse, dyspareunia) or occurs continuously, albeit pain can also be absent [2].

The way infertility is connected to endometriosis is complex and differs from one woman to another. First, infertility could be linked to endometrial dysfunction, leading for instance to implantation failures [3]. Ovarian follicles of the endometriotic women could themselves be less able to undergo normal zygotic development. This could be due to a specific accumulation of inflammatory molecules in the oocytes of endometriotic women. Their number could also be reduced, especially by age, or surgery. A recent metabolomic study using proton nuclear magnetic resonance (^1^H-NMR), compared 50 patients with Deep Infiltrating Endometriosis (DIE) versus patients with tubal obstruction infertility as control, and highlighted a molecular composition pointing to mitochondrial anomalies and oxidative stress [4]. Finally, pelvic inflammation, caused by the presence of endometriotic lesions, may impair spermatic progression throughout the fallopian tubes and fecundation [3]. 

**Figure 1:**
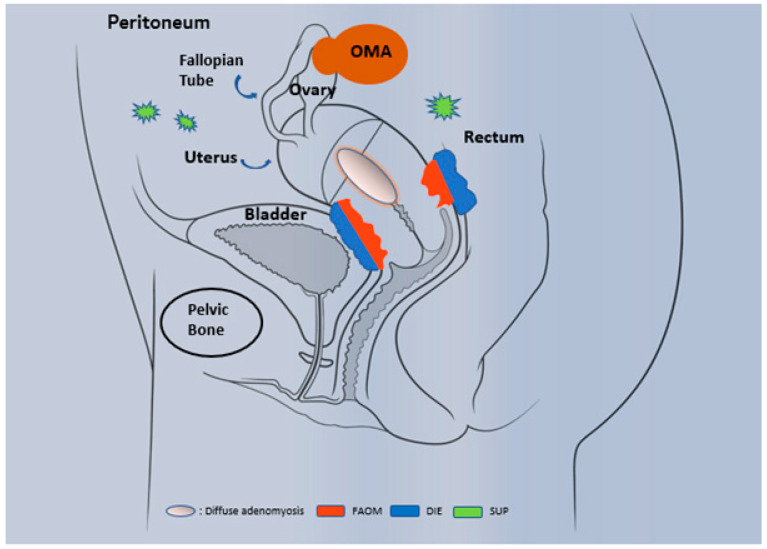
Phenotypic description of endometriosis and adenomyosis. Three well-recognized phenotypes occur in endometriosis: superficial peritoneal lesion (SUP), ovarian endometriomas (OMA) and deeply infiltrating endometriosis (DIE). In specific cases, the invasion of endometrial tissue towards the uterus leads to the establishment of adenomyosis, a specific entity that differ from endometriosis and present different forms: diffuse adenomyosis and focal adenomyosis of the outer myometrium (FAOM).

Histologically, endometriosis is characterized by the presence of endometrial glands and stromal tissue that develop as endometrium-like structures outside the uterus. Generally, the organs affected are the ovary (endometrioma OMA), the peritoneum (superficial endometriosis SUP), the retroperitoneum and the anatomic structures located near the uterus, as for instance: uterosacral ligaments, bladder, rectum and ureters (Figure 1). In addition, the lesions can affect the myometrium and then constitute adenomyosis lesions that share many features with ‘classical’ endometriosis, and were once called *endometriosis interna*.

Three well recognized phenotypes occur: superficial peritoneal lesions (SUP), ovarian endometriomas (OMA) and deep infiltrating endometriosis (DIE). OMA are cystic masses that arise from ectopic endometrial tissue and grow within the ovary. DIE, is defined as subperitoneal lesions that penetrate deeper than 5 mm under the peritoneal surface (such as the uterosacral ligaments) or as lesions that infiltrate the muscularis propria of the organs that surround the uterus (for example bladder or intestine). Less frequently, endometriosis can occur at extragenital locations. Endometriosis is stratified by the American Society for Reproductive Medicine (ASRM) classification into four stages (I, II, III and IV) according to surgical evaluation of the size, location, severity of endometriotic lesions (superficial or deep) and the extension of adhesions [5,6]. Evidence suggests that endometriosis is a stable disease (as opposed to a proliferative disease such as cancer) that progresses to fibrosis over time [7,8,9,10].

### 1.2. Diagnosis

Endometriosis is difficult to diagnose for several reasons [11]. One of the factors is probably a lack of understanding of the disease by health-care professionals. Furthermore, uncertainty exists regarding the pathogenesis of endometriosis. The heterogeneity of the disease, with three endometriosis phenotypes, and the possibility of asymptomatic disease as well as the potential comorbid presence of adenomyosis can complicate diagnosis. History-taking by patient interviews is essential for diagnosing endometriosis [11]. Pelvic pain is the cardinal symptom of endometriosis in different forms (for example, dysmenorrhea, dyspareunia or chronic pelvic pain), with the potential for overlapping symptoms [12]. Moreover, such pain can be associated with non-gynaecological symptoms (particularly urinary and/or digestive) [12,13].

Of note, the cyclic nature of the pain is a key feature of the disease [14]. Moreover, during clinical examination, health-care professionals should check for the following abnormalities: visible bluish lesions on the vaginal fornix; palpable sensitive nodules. However, a normal physical examination does not rule out endometriosis [15] while physical examination during menstruation may improve detection, but is not any more used today [16].

Medical imaging has led to substantial improvements in the diagnosis of endometriosis. Importantly, transvaginal ultrasound (TVUS) and MRI are not only suitable for diagnosing the disease but also to distinguish at least two phenotypes of endometriosis (OMA and DIE) [17,18,19,20]. TVUS should be the first-line imaging approach for the evaluation of suspected endometriosis [21,22]. Notably, SUP could be missed by imaging since the size of the lesions are below the threshold for detection [23]. Laparoscopy is no longer recommended when the aim is only to diagnose endometriosis. This strategy makes it possible to avoid unnecessary diagnostic laparoscopies. An updated vision of endometriosis diagnosis tends to consider that history-taking and medical imaging are sufficient to propose a therapeutic strategy [11]. 

### 1.3. Origin and Genetics

The origin of endometriosis lesions are discussed, but the most classical hypothesis has been posited by J.A. Sampson in 1927 [24], who, besides, coined the term ‘endometriosis’. In this hypothesis, retrograde menstruations are the source of the ectopic lesions. Today, it can be interpreted as the presence of progenitor cells in the retrograde menses that ‘memorized’ the uterine development program, and that will be able to ‘restart’ it at ectopic positions. Other hypothesis (metaplasia, lympho-vascular emboli) are possible, and some of them are summarized in [2]. Despite all its explanatory advantages, and mostly the fact that it is the one and only theory explaining the anatomic distribution of the lesions, the Sampson’s theory entails some specific difficulties. For instance, while it is estimated that >90% of women have retrograde menstruations, only 10% develop endometriosis lesions, suggesting that specific mechanisms differentiate the patients. Also, in rare cases, endometriosis lesions occur in organs located above the diaphragm, such as the lungs or even the brain [25]. 

The heritability of endometriosis has been estimated at 50%, indicating that genes are an important explanation of the disease etiology [26,27]. Identifying such genes and gene variants is a prerequisite for understanding the physiopathology and paves the way to a personalized medicine approach. Finding these genes remains a considerable challenge for many complex human diseases.

Seemingly, the most straightforward approach to identify such genes is to analyze the variation occurring in candidate genes, genes of which function is supposedly associated to the fields of the pathology, estimated at large. Another approach will use a familial positional cloning approach: the idea is to screen families with polymorphic markers covering the chromosomes, these markers having been for a long time microsatellites, are now almost completely supplanted by Single Nucleotide Polymorphisms (SNPs), which, while harboring a much lower PIC (Polymorphic Information Content), are considerably more numerous (the human genome is estimated to encompass several tens of thousands of dinucleotide repeat microsatellites, while SNPs account by the millions). For about ten years now, the drop in genotyping costs (less than $0.0001 per SNP in 2020), triggered the idea of applying these approaches to non-familial situations, leading to the concept of GWAS (Genome-Wide Association Study), where two large populations (cases versus controls) are compared statistically for a large set of SNPs (in the range of one million). Finally, the plummeting costs of sequencing drove the idea of sequencing systematically the genome or at least the exome of individuals and comparing them, generally rather inside families with cases and controls. The present review aims at comparing the GWAS and the exome-sequencing approach in endometriosis, evaluate their complementarity, and attempt to present further directions of work that should enable the practitioner and the scientist to better cope with the complexity of endometriosis. Besides SNP accessible in GWAS, sources of variations are Copy Number Variation (CNV), and indeed three CNV were reported as associated to endometriosis but will not be the topic of this review [28,29].

## 2. Procedure of This Review for the GWAS and Overview of the Results

Here, we review of GWAS in endometriosis published in the PubMed database, considering the following descriptors: endometriosis and (“polymorphism” or “SNP” or “genetic polymorphism”) and (“GWA” or “Genome-wide” or “Genome wide” or “Genetic association study”).

The inclusion of articles in this review was carried out according to the following criteria: (i) endometriosis GWAS based either on a case-control or meta-analysis study design, (ii) studies published in English language, (iii) publications with available full-text and (iv) studies published until April 2021. The exclusion criteria were: (i) publications with no GWAS but performing the analysis of candidate polymorphisms in endometriosis and (ii) review studies. The list of publications used is presented as Table 1.

After the selection of the manuscripts and complete reading, the following information were collected: author; publication year; study type; cohort of study; number of cases and controls; cases and controls sources; inclusion and exclusion criteria of cases and controls; most associated SNPs; risk allele frequency (RAF) data; and endometriosis association data. Chromosomal location, dbSNP ID, locus, position (bp), risk allele, gene and SNP location in the gene were collected from the studies included in this review or from the dbSNP database (www.ncbi.nlm.nih.gov/snp/, accessed on 31 May 2021). 

Initially, 102 publications were found through the search strategy previously mentioned. After reading the titles and abstracts, 64 studies did not perform a GWA study in endometriosis, 8 review studies and 10 were replication studies leading to remove 82 articles. Finally, 20 articles were included in this review, of which 9 were case control and 11 were meta-analysis studies. The other studies that did not satisfy our criteria however are relevant for endometrioses are discussed in the text. Overall, 44,612 endometriosis cases and 247,145 controls were analyzed. The number of participants in each study was quite different (171 to 17,045 for the cases and 308 to 150,021 for the controls, note that this addition gives only a rough estimation, not considering for instance the ethnic background that was generally similar for cases and controls in a given study), with a predominance of European ethnicity followed by Japanese origin. Most endometriosis cases were either diagnosed by laparoscopic surgery (not systematically histologically confirmed) but also often self-reported [13,16,18,19,20,23,37]. In 2015, Borghese and coworkers [37] categorized endometriosis in SUP, OMA and DIE, and in 2017 Wang and coworkers [39] used only women with OMA. Only in the Uno and co-workers study in 2010 [30] was considered as a diagnosis method the multiple clinical symptoms, physical examinations and/or laparoscopic surgery; however had no information about endometriosis stage. The selection of the control group was different among the case-control studies: women with other diseases [30,39]; women with negative diagnosis of endometriosis after surgery [35,37,39]; women with negative diagnosis of endometriosis by magnetic resonance imaging (MRI) [35] or who had declared themselves as healthy women [42]; and women who had no evidence of endometriosis diagnosis; however did not report negative diagnostic criteria [40].

About 47% performed only one stage (discovery stage) and 53% performed both the discovery and replication analyses. The number of SNPs identified within each study varied between 2 and 22, being Pagliardini and coworkers in 2013 [34] the study with the least number of SNPs associated with endometriosis and Sapkota and coworkers in 2017 [41] and Sobalska-Kwapis and coworkers the same year [42], the two with the highest number of identified markers. 

Fourteen genes/ SNPs were associated with endometriosis risk in more than one article (chromosome 1, 2, 6, 7, 9 and 12; WNT4, GREB1, FN1, IL1A, ETAA1, RND3, ID4, NFE2L3, CDKN2B-AS1, VEZT, SYNE1, FSHB, ESR1, ARL14EP). SNPs were localized in intergenic and intronic regions their risk allele frequencies varied among the studies and their results were conflicting.

The exome analyses are described independently and did not request a specific “meta-analysis-like” approach, given their limited number.

## 3. Genome-wide Association Studies

A list of significant SNPs from the different studies is provided as Table 2.

### 3.1. Large GWAS from Heterogeneous Populations

The 10 most robust SNPs found from the different large GWAS in endometriosis have been described and discussed in detail in 2020 [49]; here we present an additional discussion of these studies. The first large-scale GWAS on endometriosis was carried out in 2010 in Japan [30]. This study was based upon 1907 cases and 5292 controls and revealed an association between CDKN2B-AS1 and endometriosis, on chromosome 9p21, and a trend towards association with WNT4, a gene of the WNT-Beta catenin cascade previously directly involved in female sex determination [50]. A second one starting from Caucasian women (Australia and UK) with 3194 cases and 7060 controls led to the discovery of rs12700667 on chromosome 7p15.2, in the intergenic region located between NFE2L3 and HOXA10 (OR = 1.22) [32]. It was followed one year later by a meta-analysis [33]. As early as 2010, a meta-analysis was also undertaken for 696 patients and 825 controls [31]. At *p* < 10^−5^, several SNPs were found inside and nearby the gene IL1A, and downstream of RHOU (Ras Homolog Family Member U); despite the limited size of the sample set in this paper, at the threshold chosen, there was a slight increase in the number of putatively significant SNPs compared to that expected by mere chance (36 vs. 28). The increased size of later meta-analyses allowed to find systematically SNPs that were robust enough to reach genome-wide significance (generally established at *p* < 5 × 10^−8^ to consider for multiple testing) such as published by Sapkota and coworkers in 2017 [41]. 

The metanalysis of 11 GWAS of endometriosis has been performed in 2017 [41] and made it possible (through the mechanical increase in sample size: 191,596 controls and 17,045 endometriosis cases) to enrich the list of genes with five additional loci (FN1, CCDC170, ESR1, SYNE1 and FSHB), leading in 2017 to a list of 19 SNPs associated with the risk of endometriosis at the genome wide level (i.e. a *p* value below 5 × 10^−8^). For the five novel genes, the relative risks were 1.06, 1.09, 1.10, 1.15, and 1.11, respectively. When the analysis was carried out on stage III-IV endometriosis, the relative risks were comprised between 1.15 and 1.35. Again, the effect of each variants explained only a small portion of the variance. 

### 3.2. GWAS Studies from Genetic Isolates

Amongst the large GWAS studies, starting from partially consanguineous populations is an interesting alternative, since isolation and genetic drift may lead to cumulate the advantages of linkage studies in families with those of large-scale GWAS based upon the genetic analyses of populations. As such, the Icelandic population has been a paradigmatic tool for decrypting the fundamental genetic bases of single gene as well as polygenic diseases, with the systematic collection of DNA samples from the entire population that was maintained at less than 50,000 individuals for more than 1000 years [51]. An inconvenient would nevertheless be that the genes found may be specific to a limited number of populations and may not have a general interest for the pathophysiology of the disease. One large study of endometriosis was undertaken in the Iceland population [52], with 1840 cases and 129,016 control women. Interestingly, 9 out of 11 of the loci previously identified from the previous published GWAS at this date, were confirmed at a nominal *p*-value of *p* < 0.05, far below the Genome-Wide significance, but suggestive as replication findings (and thus based upon much less multiple testing, WNT4, GREB1, ETAA1, NFE2L3 [2 SNPs], CDKN2B-AS1[2 SNPs], VEZT), while RND3 and ID4 could not be confirmed in this 2016 study. Besides, three genes were found in addition to this short list: KDR (OR = 1.24), TTC39B (OR = 1.28), and RTN4RL1 (OR = 9.419). In these three cases, very interestingly, the association with the severest cases of endometriosis (stages III and IV, about half of the samples) was stronger (1.47, 1.35 and 15.45, respectively). The case of RTN4RL1 is particularly interesting, with the strongest relative risk found from all these GWAS analyses. The variant corresponds to a single base pair deletion (rs767233639, MAF = 0.05%) located in the 3’ UTR of the gene. RTN4RL1 encodes a receptor for the chondroitin sulfate proteoglycans. The mechanisms linking this gene with endometriosis have not yet been deciphered. Despite the risk variants being extremely rare, understanding this aspect could be an inspiring research track for future investigations in the pathogenesis of endometriosis. 

The Sardinian population is another isolated human population of particular interest for genetic studies, allowing theoretically to find relevant associations with a limited number of samples. In the Angioni study [53], the DNA of 72 women was collected (41 with symptomatic endometriosis and 31 controls). Despite this limited sample size, the authors could confirm a significant effect for previously described VEZT variant at the homozygous state, the CC allele being apparently protective (*p* = 0.0111, OR = 0.0602, CI (0.005–0.5601)). A more systematic search for coding variants (11) of VEZT was undertaken in Australian women in 2016 [54], in connection with the expression of this gene (located in 12q22). The level was also examined in endometrial glands, showing an increase connected to the secretory phase of the uterus in the glandular epithelium. One of the SNPs (rs10859871) acts as an expression quantitative trait locus (cis-eQTL) on the VEZT gene, being associated to an enhanced expression of the gene, as well as to the regulation of NR2C1.

### 3.3. Small-scale GWAS and Pooling Approaches

Curiously, besides the large scale GWAS carried out from tenths or hundreds of thousands of DNA samples, some small-scale studies were performed with only hundreds of DNA samples, but that may be interesting as based upon specific populations or specific phenotypic criteria. For instance, in 2017 Sobalska-Kwapis and co-workers published results based upon 171 patients and 2934 controls from Lodz Hospital (Poland). The authors identified 22 significant SNPs with an adjusted *p*-value < 0.05, starting with 274,400 filtered SNPs. On chromosome 6, a series of SNPs in strong linkage disequilibrium were associated to endometriosis, especially in C2 and HLA-DRA. Strikingly, none of the previously found 22 SNPs from larger scale studies and other previous studies [34,35,41,55,56,57,58,59,60] could be retrieved in this specific paper. Worse, in this case, the unadjusted *p* values were marginally significant (0.004 < *p* < 0.05) only for three of them (rs2235529-WNT4, rs6907340-LOC100506885, and rs10757272-CDKN2B-AS1). Another approach theoretically necessitating less samples is the pooling, aiming at evaluating allele frequencies according to the signal intensities on SNP microarrays, such as published by Borghese and co-workers [37]. In this study, the initial pool for discovery was composed of two pools of 10 patients affected either by OMA (ovarian endometrioma), SUP (superficial endometriosis) or DIE (Deep Infiltrating Endometriosis). The identified SNPs were then tentatively replicated at the individual level on a cohort of 259 endometriosis and 288 controls. In this study, all the diagnoses were systematically histologically validated, and homogenized for BMI, parity, gravidity, and infertility. This led to the identification of rs227849 near RUNX2, rs4703908 near ZNF366, rs2479037 in VTI1A, and rs966674, in an intergenic region of chromosome 5 located between LOC107986436 and LINC02113, as connected with OMA. A pooled approach of Brazilian samples pointed out to KAZN and LAMA5 (394 infertile women with endometriosis and 650 fertile controls) and was reproduced in a relatively limited number of human samples [45]. A third pooling experiment was published in 2017 [39]. In this last study, a case-control approach was performed allowed the identification of 10 additional susceptibility loci, three of them reaching a *p* value of 5 × 10^−6^ (nearby IGF1R, C7ORF50 and MEIS1).

### 3.4. Genetic Connections between Endometriosis and Other Diseases and Phenotypes

Since endometriosis symptoms are not specific (pain, infertility), one can hypothesize that the underlying genetic risk factors would be shared with other diseases (albeit this may also be due to shared environmental effects). This has been recently explored by crossing GWAS analyses for other phenotypes, especially pregnancy disorders, including infertility manifestations, such as Recurrent Implantation Failure (RIF) or Recurrent Pregnancy Loss (RPL), as shown in a recent meta-analysis of gene profiling studies [61]. There are common alterations of endometrial functions, such as cell-cycle alterations, but also in ciliogenesis, and in RIF and RPL anomalies of expression of genes involved in mitochondrial function. While this study is not a GWAS, it shows that common deregulations could be at work to explain at least partly the connections between infertility and endometriosis.

Endometriotic women are often presented as leaner that other women [62]. Nevertheless, Obesity (that given this observation could be considered as protective), which was found as a causal risk factor in uterine endometrial cancer as shown through Mendelian Randomization [63], was not directly associated (negatively or positively) with endometriosis, and gene expression alterations did not differ between obese endometrial women and others [64]. The association between endometriosis and maternal BMI has also been addressed through the analysis of common susceptibility loci [36]. This showed that there is an overlap between endometriosis and fat distribution evaluated for instance, by the waist-to-hip ratio adjusted for BMI, and allowed the identification of novel loci near KIFAP3 and CAB39L, and shared genetic basis for WNT4, GRB14 and the intergenic region 7p15.2. The associations were stronger with stage III-IV endometriosis. Another study based upon Mendelian Randomization by Two-Sample comparison attempted recently to identify associations of specific biological parameters with endometriosis, using three methods (weight median-WM, MR-Egger-MRE, and Invers Variance Weight-IVW). The only situation where the three methods were congruent is with the trait ‘length of the menstrual cycle’, associated to a reduced risk of endometriosis (between 0.1 to 0.38) [65]. Besides, significant SNPs linking endometriosis with phenotypes were found for sex-hormone levels, age at menopause, at menarche and again, the length of the menstrual cycle.

Amongst the unexpected associations that have now been found through the cross analysis of various GWAS data, endometriosis and depression were found to share similar risk loci, linked with gastric mucosa abnormality [66]. Other associations link Endometriosis with migraine [46]. In this case, the analysis of concordant SNPs revealed highly significant overlaps, especially for the 1% most significant SNPs together present in Endometriosis and Migraine (85) compared to those that were discordant (43), revealing a 3.61 Odds Ratio compared to the null hypothesis (*p* = 7.2 × 10^−4^).

Leiomyoma is a benign tumor of the muscular tissue of the uterus that is now strongly associated to endometriosis [67]. Somehow, Leiomyomas are like adenomyosis lesions, where the lesion occur through the uterine wall, rather than from material transiting through the Fallopian tubes. The systematic analysis by GWAS of leiomyoma-associated variants was undertaken in 2019 [48] starting with 16,595 cases and 523,330 controls, and led to the identification of 21 variants in 16 loci associated to the disease. In 208 patients with symptomatic leiomyoma, histologically proven, 181 had concomitant endometriosis lesions. More recently, a GWAS analysis including 35,474 leiomyoma cases and 267,505 controls identified 8 loci with a genome-wide significant level (*p* < 5 × 10^−8^), adding up with the 21 reported loci. Four of these loci are also significantly associated with the risk of endometriosis at 1p36.12 (LINC00339-WNT4, rs7412010, OR (95% CI) = 1.13 (1.11–1.16), *p* = 2.43 × 10^−29^ [35,55]), 2p25.1 (GREB1, rs35417544, OR (95% CI) = 1.09 (1.07–1.10), *p* = 2.32 × 10^−19^), 6q25.2 (SYNE1-CCDC1170 rs58415480, OR (95% CI) = 1.19 (1.17–1.22), *p* = 1.86 × 10^−54^), and 11p14.1 (FSHB, rs11031006, OR (95% CI) = 1.10 (1.07–1.12), *p* = 5.65 × 10^−15^). 

A recurrent important question is that of the putative association of endometriosis and cancer, especially endometrial and ovarian cancer. The exhaustive study of Painter and coworkers [44] identified 13 loci associated together with endometriosis and endometrial cancer, with *p* < 10^−4^ (PTPRD, PDZRN3-CNTN3, SKAP1, KITLG-DUSP6, TFAP2D, KLF3-TLR10, LMO7-KCTD12, PARP11-CCND2, ZNF536-TSHZ3, THEM215-APTX, CEP112, WNT4-ZBTB40, PRIM2). Despite this finding, the relatively limited statistical significance of these results do not point to endometriosis as being a strong risk for endometrial cancer, which is anyway not documented by epidemiology. The association between cancer and endometriosis has been addressed systematically in [68], strengthening the idea of an association with clear cell ovarian carcinoma (OR = 3.44), endometrioid cancer (OR = 2.33), thyroid cancer (OR = 1.39), and marginally to breast cancer (OR = 1.04), while no association was found with endometrial cancer, and other cancers. In terms of genes actually found, only the region of WNT4 was identified alone in common with the endometriosis GWAS, and only one SNP reached genome wide significance, rs2475335 in PRPRD. PTPRD is a protein tyrosine kinase involved in many basic cellular processes linked to cell growth and division. Concerning the genetic link with ovarian cancer, the top 38 endometriosis-associated SNPs identified in the Nyholt study in 18 regions were tentatively associated with this type of cancer [44]. Three ovarian cancer GWAS were collected (15,361 ovarian cancer cases and 30,815 controls), leading to the identification of 8 SNPs from five chromosome regions. The strongest burden statistic was on chromosome 1p36 (the region encompassing ZBTB40, WNT4 and CDC42), with two types of ovarian cancer (clear cell carcinoma and high-grade serous carcinoma, while epidemiologically, endometriosis does not constitute a risk factor for this last type of cancer). The potential link between endometriosis and ovarian clear cell carcinoma has been documented before, with common deregulation in protein expression, such as PTEN, which is decreased in both diseases [69]. Interestingly, a Loss Of Heterozygosity (LOH) was detected in the PTEN region at 10q23.3 in endometriosis lesions, which revealed somatic variants, as well as preexisting germline variants, putatively associated with decreased expression and development of a common risk situation for ovarian cancer and endometriosis [70]. In fact, the occurrence of somatic mutations leading from endometriosis to cancer is now well documented for the important epigenetic regulator ARID1A, but of course, in the context of genetic predisposition, events of the germinal lineage are the only one considered in the present review [71]. 

Another recent paper attempted to link gynecologic diseases and endometriosis in the Japanese population without finding significant SNP in endometriosis probably due to the limited sample size of endometriosis cases in this study [72]. The other gynecologic diseases analyzed include fibroids, ovarian cancer, and uterine endometrial cancer, which in this study allowed to identify significant SNPs, despite relatively limited sample sizes, as well. 

In 2016, a study by Horikoshi and coworkers [73] tried to connect birth weight-influencing genes (60 genes identified) with various human diseases or parameters, such as blood pressure, diabetes, coronary heart disease, but also endometriosis. Even by far, none of these genes passed genome-wide threshold (~5 × 10^−8^), suggesting that birth weight is not connected to the risk of developing endometriosis later. A similar GWAS study aiming at identifying gene of reproductive behaviour failed as well to be found in common as at risk for endometriosis, albeit GATAD2B and ESR1 were found as most likely causal and are indeed increased in expression in endometriosis lesions compared to the eutopic endometrium [74]. The ESR1 region is well-known as associated with reproductive disorders, genes of the ESR1 region are correlated with ESR1/PGR genes expression level and PG concentration [75].

Another study determined associations between endometriosis significant GWAS SNPs and other reproductive phenotypes [76]. This study found significant SNPs associated with dysmenorrhea that were also identified in endometriosis. This was true for CCDC170-ESR1 (rs6557160), a locus previously identified with the SNP rs1971256 by Sapkota and coworkers [41] and confirmed IL1A (rs10167914) [38] and ID4 (rs7739264, rs760794) [55]. 

Recently, in the Iceland population, Olafsdottir and coworkers [77], revealed an association of rs3820282 (in an intron of WNT4, probably encompassing the response to estrogen signaling) with Pelvic Organe Prolapse, thus allying this gene with endometriosis, leiomyoma, gestational duration, and as mentioned above, up to the early stages of female sex determination [50].

These results attempting to connect endometriosis with other diseases led therefore to various results in term of finding actual intersections (strong for leiomyomas and weak for gynecologic cancer predisposition). This suggests that a clear phenotypic definition is warranted to have GWAS that perform better in finding relevant genes. As a mirror vision, it clearly suggests that ‘endometriosis’ is rather a compendium of symptoms having similar manifestations, hiding subtle, different and maybe complementary subjacent genetic causes (as gene or genome variations).

### 3.5. Genetic Regulation of Gene Expression and System Biology Analyses in Connection with GWAS Approaches

In complex traits and complex diseases, variants associated to alterations of gene expression located either nearby the gene with its expression modifed (in cis) or at long distances or even from other chromosomes (in trans) have been systematically studied when expression data were available together with the genotypes. In endometriosis as well, several studies made the link between the GWAS and eQTL. One recent study in Taiwan [78], presented novel candidate genes (PTPRD on chromosome 9 and two other non-coding regions at chromosomes 14 and 15). A cis-eQTL approach was carried out in the same study pointing to the expression regulation of INTU via rs13126673. Associating expression profiling with SNP also led to the identification of eQTL and this information, collated with the position of genes identified by GWAS, allowed to connect genetic variation and classify the patients according to gene expression levels and to find eQTL located in cis or in trans (45,923 and 2.968, respectively, corresponding to 417 genes and 82 genes, respectively [79]). In this paper, the association between endometrial eQTL signals (associated with expression alterations during the menstrual cycle) were tentatively connected with endometriosis, but also with PCOS and endometrial cancer.

Recent progresses in the determination of gene/protein cascades specifically altered in disease is a novel ‘system biology’-based approach to enrich the knowledge database, for instance in endometriosis. Interestingly, a 2017 study revealed a relatively strong association of rs144240142, inside the MAP3K4 gene (OR = 1.71), but specifically with the mildest forms of the disease (rAFS I/II), and MAP3K4 was differentially expressed according to the stage of the endometriosis. This signalling cascade plays multiple roles in cell physiology (cell division, gene expression, cell movement and survival), and was not pointed out before despite the relatively limited size of the experimental setting (3194 cases and 7060 controls), compared to the original dataset encompassing almost 200,000 controls [40].

## 4. Replication Studies

In many cases of complex diseases, it appeared that SNP found in a discovery analysis failed to be replicated in smaller studies. Among the major recognized causes of this observation is the ‘Winner’s curse’ effect, an overestimation of the effect of a given SNP that is significant either because it is really associated to the locus found, or is ‘lucky’ in the context of the DNA sample tested, especially if its size is relatively limited. Another frequent cause is the genetic heterogeneity of the population under study. In terms of SN¨Ps, some variants may be polymorphic in some populations and monomorphic in others, leading to non-replicability [80,81]. Endometriosis is not exceptional to this respect. In 2015, Sapkota and co-workers tested 10 loci previously found for a replication attempt based upon 998 confirmed cases and 783 disease-free controls. Three coding variants in GREB1 and CDKN2B-AS1 were nominally associated with the risk of endometriosis [82]. Soon after the primary publication, the meta-analysis of Pagliardini and co-workers confirmed the involvement of three SNPs out of four tested, rs1333049 (CDKN2B-AS), rs7521902 (close to WNT4), rs125048 (FN1, specifically for the most severe forms), while rs127800667 (intergenic of 7p15.2) was not significant (OR = 1.00). Interestingly an epistatic positive interaction was discovered for rs7521902 and rs1500248, leading to a OR of 2.15 for OMA [34]. This study used 305 endometriosis patients and 2710 controls. A similar study was carried out in the Chinese population [56], based upon 580 patients and 606 matched controls and confirmed the association between rs12700667 (intergenic region at 7p15.2) and endometriosis. This association between rs12700667 at 7p15.2 and endometriosis was not re-identified in all of the replication attempts [34,83], suggesting differential effects according to the populations considered. The other SNPs of this study were in CDKN2BAS and LINC00339-WNT4, but did not reveal significant associations, which is in mirror with the previous study. WNT4 was further confirmed in 400 Brazilian infertile women with endometriosis, compared with 400 control fertile women [84]. An early replication study [83], based upon the analysis of 1129 patients and 831 controls, validated only rs1250248, in the FN1 gene, out of four SNPs analyzed (the three others being rs7798431 and rs12700667 in an intergenic chromosome 7 region, and rs7521902 - LOC105376850). Concerning IL1A that was initially pinpointed through an early meta-analysis [31] with a *p* value as high as 10^−5^, the locus involvement was later confirmed in a different population for 8 different SNPs [38] and another population of Japanese origin [85] for four SNPs in linkage disequilibrium. The Sapkota validation of IL1A [38], led to a significantly confirmed association for rs6542095, but with a still moderate OR (1.21). Another validation was performed in a population of Iranian women (105 cases, 102 controls) [86]. Three SNPs were analyzed and rs2856836 allele C was found increased in endometriotic women in this population as well (OR = 2.2).

Osinski et al attempted to validate the GWAS results for 10 significant SNPs from the seminal analyses on a cohort smaller than the ones initially used (315 endometriosis and 406 healthy fertile women as controls) and could overall confirm rs12700667 near NFE2L3, and rs4141819 near ETAA1, for the infertile women with stages III/IV [47]. In the initial study [55], the OR associated with rs4141819 was estimated at 1.22 for the risk allele, and at 1.20 for rs12700667. Overall, the 10 SNPs present with calculated relative risks comprised between 1.12 and 1.24, therefore the two that were confirmed in the Osiniski series are not specifically the ones with the highest OR. It can be estimated that the primary discovery of the 10 SNPs relies enormously on the initial sample size and that the one of the Polish study is under the threshold that would allow to confirm all of them. The same type of approach was used for an Italian (Sardinia) population in 2020 [53], where the authors analyzed variants located in WNT4 (rs7521902), VEZT (rs10859871) and FSHB (rs411031006). 

**Figure 2:**
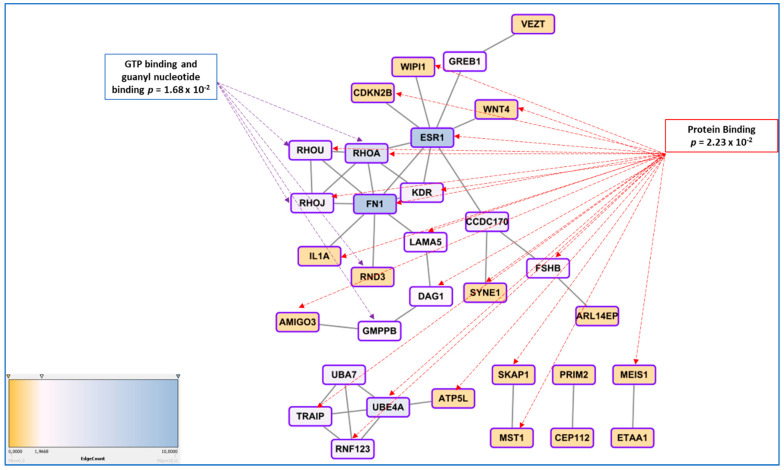
A significantly enriched gene network is constructed from the list of genes found through GWAS approaches using the online tools STRING and Cytoscape, that allowed to identify hubs (genes with the highest degree of connection, ESR1 and FN1). Functional annotation using BINGO led to discover the most enriched molecular functions, connected to GTP metabolism and Protein binding, in a broad sense. The Edge count associated with the color is proportional to the number of links connecting the different genes.

To attempt identifying a general message in the genes found through GWAS approach, Albertsen and Ward suggested that at least 4 genes found by this approach relate to the regulation of the actin cytoskeleton [87]. Here we present a more thorough analyses using gene network tools. To compute the network presented in Figure 2, we provide the list of genes to the String online tool available at https://string-db.org/ (accessed on 31 May 2021), as human proteins, allowing the software to identify relations between the genes based upon text-mining of the literature, experimental demonstrations of physical interactions, co-expressions, leading to establish the network and calculating scores for couples of genes (in this case the parameter for confidence was by default the medium level, corresponding to a score > 0.4). In the specific case of our dataset, for each couple of proteins, the score varied from 0.4 (for instance FN1-RHOU) to 0.98 (GREB1-ESR1). The network structure per se was significant with 36 connections (edges) found while only 18 were expected at random (*p* = 0.000148).

**Figure A1:**
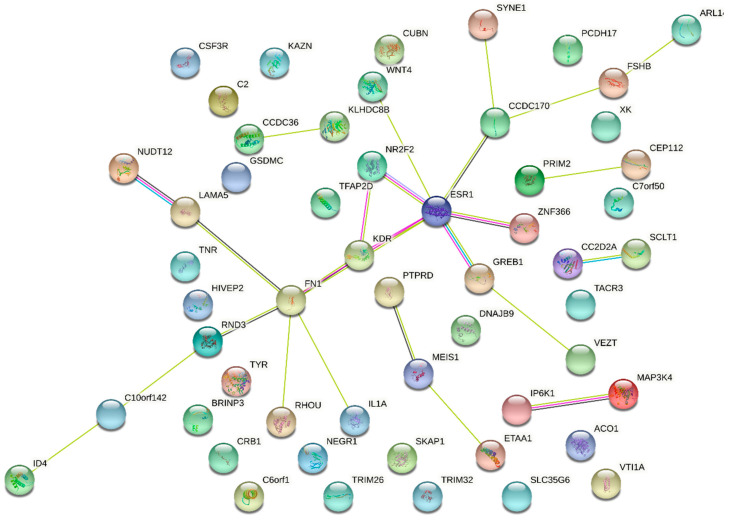
The complete network analysis by String of the genes found in the different published endometriosis GWAS.

The network obtained was exported to Cytoscape as a TSV table (https://cytoscape.org/ (accessed on 31 May 2021)) to explore the possible hierarchical relationships inside the network, strengthening the role of hubs for ESR1 and FN1 (data presented from the String database and Cytoscape software, https://string-db.org/ (accessed on 31 May 2021) and https://cytoscape.org/ (accessed on 31 May 2021)). Figure 2 presents the part of genes that are connected with ESR1 and FN1 (and not the genes that are not connected with this main network or smaller ones; the complete network is presented as Figure A1).

Analysis of GO terms showed several significant enrichments, in particular for cell-junction (10 genes: ARL14EP, SKAP1, RHOU, RHOA, RHOJ, KDR, DAG1, VEZT inside the network and GABRA1, CABP1 and KAZN, outside). Besides the GTP binding association, a group of genes involved in proteasome function was also enriched (UBA7, UBE4A, TRAIP, RNF123, *p* = 0.0068).

## 5. Functional Studies

Finding significant SNPs using GWAS approaches is currently straightforward, given the huge number of available SNPs that can be genotyped simultaneously (in the million range), given a collection of DNAs is available from enough control and cases. Nevertheless, these approaches led to two facts:

- In endometriosis, as in many complex diseases, there is a discrepancy between the calculated heritability and the sum of the SNP effects found in GWAS. This difference is explained by the concept of missing heritability [88]. More precisely, it has been estimated in 2012 that the total variation tagged by frequent SNPs as used in GWAS was 0.26, i.e., about half of the total genetic variation [55]. Missing heritability is currently explained by various hypotheses, one of the most prominent relies in the idea that GWAS are carried out using microarray platforms encompassing SNPs having a relatively high Minor Allele Frequency (MAF) and hence, will miss rare alleles that may be the one indeed associated to the disease. These rare alleles have presumably been counter-selected through the mechanisms of evolution. An indication of such possibility is provided in [41], where the authors focused on protein modifying variants analyzing 7164 cases and 21,005 controls for the discovery and 1840 cases and 129,016 controls in the replication cohort. The only locus that was replicated was *GREB1* at2p25. Even in exome arrays designs, rare variants are not massively present, which could explain this relative failure. The authors logically conclude that sequencing high-risk families at the exome level is a promising way to identify novel rare variants in genes involved in endometriosis. Another major issue is the impact of inter-patient variability in the big GWAS approaches, suggesting that optimizing the clinical classification of the patients could improve the detection power of the GWAS [89,90]. Other explanations are the possible association of disease with CNVs (variations in length of large repeats), which are poorly addressed by classical arrays, epigenetics regulation that may also mask some of the gene-defined variation, as well as epistatic mechanisms that implies that for obtaining a tangible phenotype, the co-occurrence of two or more gene variants is requested [91].
- Finding a significant SNPs does not explain how, functionally, this SNP triggers the disease risk, the situation being made even more difficult because the relative risk is generally comprised between 1.1 and 1.5, meaning that many carriers of the ‘at-risk’ variant are not stricken by the disease, while carriers of the ‘protective’ variant are not at all. This latest question was systematically addressed in a 2015 review by Fung and co-workers, and showed that once the variant is identified, a long and tedious stroll commences, from a refined mapping with additional SNPs in the region identified, a study of existing functional annotations (which is difficult when non-coding or intergenic SNPs are found, a case encountered for the chromosome 7 rs12700667 in endometriosis ), a measure of cell-type specific gene expression and protein levels, analysis of the cell-type specific local epigenetic regulation, cell models and animal models, eventually, if available, a complicated issue in the case of a human-specific disease such as endometriosis [92].

In this context, vezatin (VEZT) has been validated in several replication studies through the validation and further analyses of rs10859871 and rs14121 SNPs. In 2016 Holdsworth-Carson and coworkers demonstrated that this SNP acts as an eQTL especially in endometrial tissue of endometriosis patients, with the A allele at rs14121 apparently inducing an overexpression of VEZT, and an opposite effect of rs10859871 [54]. *VEZT* encodes a transmembrane protein localized at *adherens junctions* and bound to myosin VIIA. *Adherens junctions* genes are generally strongly up-regulated in endometriosis compared with eutopic endometrium [10], suggesting that it could contribute to a relatively low potential of development of the endometriotic lesions. This led sometimes to coin endometriosis as a benign metastasic disease, an oxymoronic formulation.

In 2015, Fung and coworkers attempt to analyze the function of GREB1 (Growth Regulation by Estrogen, in Breast cancer 1), located at 2p25.1. GREB1 protein expression was modified according to the stage of the cycle and the cell type (specifically in the glandular epithelium and not in the stroma [92]. However, the protein quantification did not reveal obvious differences between endometriosis and control patients. Several explanations are proposed by the authors, such as the presence of mixed populations in the tissue sample. Another possible reason for this could also be that the relative effects of the variants are quite small, while the techniques of Immunohistochemistry (IHC) and Western Blot (WB) may not be sufficiently resolutive to pinpoint the differences. The figures shown in the paper show a massive dispersion of the normalized signals (mRNA and proteins, probably barring the identification of statistical differences). 

**Figure 3:**
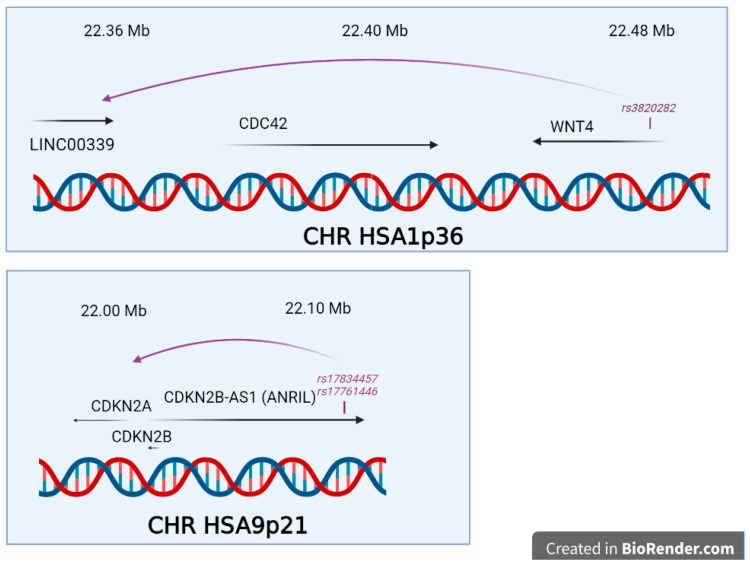
The two situations for which mechanistical insights were obtained on the functional effects of the significant SNPs. In both cases through chromatin structure approaches, such as 3C, the impact of the SNPs on adjacent genes was analysed in detail (created in BioRender.com).

In two cases, the genetic regulation explaining the QTL effect was mechanistically analysed as presented in Figure 3, at chromosomes 9p21 and 1q36. One of the most thorough mechanistic analysis in endometriosis has been published for the locus proximal to 9p21 and encompassing the gene CDKNB2-AS identified firstly by Uno and coworkers and duly confirmed later [30,34,42,52]. In their study, Nakaoka and coworkers re-sequenced 1.29 Mb in 48 individuals from Japan [93]. Amongst the 4215 SNPs and 664 indel found, they prioritized 16 SNP and 2 indels that were in total linkage disequilibrium with rs10965235 from the original study [30], and 7 SNPs and 1 indel in total linkage disequilibrium with rs1537377, 49 kb downstream [55], this one being informative in both Japanese and European populations. Using the ENCODE database, the authors crossed the genetic information with DNAse I Hypersensitivity Sites (DHS), which mark open, active chromatin, and identified two SNPs in the mid region of a cell-specific DHS (rs17761446 and rs17834457), located 76 bp apart. These SNPs are inside an intron of the large isoform of *ANRIL*, a long noncoding RNA in this region containing in addition *CDKN2A* and *CDKN2B*. By Chromosome Conformation Capture (3C), the authors demonstrated a loop interaction between the SNPs of the DHS and the promoter of *ANRIL*. Using the HEC251 and HEC265 cell lines, heterozygous for the two DHS SNPs, and a SNP in the ANRIL promoter, the authors showed that there was a preferential allele-specific interaction between the two loci, separated by ~100 kb. The DHS overlap with binding sites for TCF7L2, as well as H3K27ac, EP300 and TBP, and these interactions were validated by ChIP seq experiments, that also allowed to pinpoint an excess of binding of G versus T allele at rs17761446. In sum, the G allele appeared protective and corresponded to a 1.78-fold overexpression of *ANRIL* over the risk allele (T). Further, induction of the WNT cascade with a pharmacological treatment with CHIR99021 led to an induction of ANRIL, and to the decrease of cell cycle inhibitors (p16*^INK4A^* and p15*^INK4B^*). This link is an interesting insight, especially given the recurrent finding of WNT4 variants in the pathogenesis of endometriosis. 

At 1p36.12, there is an important locus involved in endometriosis encompassing LINC00339, CDC42 and WNT4 in a LD block of ~130 kb. Interestingly, the WNT4 locus has been associated to the development of the female sex, since duplication of the locus leads to XY female sex-reversal [94]. The locus has been robustly, and several times confirmed in endometriosis. The function of the locus has been recently addressed [95]. The SNP rs3820282, located in the first WNT4 intron, plays the role of a cisQTL strongly affecting the expression of LINC00339, in the blood and the endometrium. In the Powell study, the authors studied two putative regulatory elements located inside CDC42 (PRE2) and inside WNT4 (PRE1) through 3C approaches enabling to materialize distant interacting regions. In the case of PRE2, the insertion of specific variants at rs12038474, induced either a super-activation of CDC42 promoter activity or a decrease activity of the same promoter. This element was suspected to allow an estrogen regulation, but this was not experimentally validated [95].

In summary, the validation of SNPs is only starting in the endometriosis context. Many approaches involving genome editing, systematic sequencing, and the use of animal models or organoids [96,97] will in the future be consistently used to solve the mechanistic issues raised by the identification of the SNPs.

## 6. Exome Sequencing

### 6.1. Family Studies

This original approach allowed in 2016 to detect hemizygous deletions in two genes UGT2B28 and USP17L2 using three generation families [98]. The two genes harbour hemizygous deletions that were traced to the grandmother of the family. The first gene intervenes in reaction where glucuronic acid is conjugated to lipophilic substrates, while the second is a de-ubiquitinase and acts therefore probably in reversing the trajectory of proteins programmed to degradation by the proteasome. The ultimate validation of these approaches is terribly challenging, since proving the involvement of genes requires the use of animal models or at least strong cell biology cues from cell culture experiments. Therefore, the two loci identified will warrant further validation.

Matalliotakis attempted to evaluate five GWAS-identified variants in a familial structure (inside WNT4, VEZT, FSHB and two inside IL16). In this case, none was validated [99]. The familial structure was on three generations, with 7 women affected (one in generation I, 3 in generation II and 3 in generation III). The family appeared entirely homozygous (cases and controls) for rs7521902 (WNT4), rs10859871 (VEZT), rs11031006 (FSHB). For IL16, the risk alleles were G and T for rs11556218 and rs407211, but not at all systematically found in the patients. This data suggests that the genetic determination of SNPs through GWAS approaches may point to genes or SNPs that are not so important in familial cases. It could be hypothesized that in familial forms of endometriosis (that are in fact the basis of the estimation of heritability), the variants at risk have a major determining effect, and is located high inside the upstream cascade leading to the disease. On the contrary, GWAS points to robustly identified (often replicated) genes but that may have a marginal effect in the aetiology.

### 6.2. Population Studies

In 2014, Li and coworkers undertook an analysis of endometriosis patients from an Exome-seq approach [100]. The authors used blood, eutopic endometrium and ectopic endometrium DNA from 16 endometriosis patients, and normal endometrium from 5 healthy women. Given its limited sample size, the aim of this study was not to discover predisposing genes inherited through the germline but rather of course to identify genes that are prone to somatic mutations associated to the pathogenesis. Apparently, no overlap could be found with the GWAS-identified genes so far.

## 7. Conclusions

GWAS approaches have seldom clarified extensively the genetics underlying complex phenotypes and diseases, whatever the question addressed (human size, diabetes, cancer predisposition and so on). As a striking recent example, genetics of osteoporosis uncovered hundreds of loci, with the recent study by Morris and co-workers that identified 501 loci, 301 of which were new, and explaining only 20% of the variance when cumulated [101]. Endometriosis is no exception to this rule, while GWAS pinpointed to ~30 genes that are significantly associated to endometriosis. It is nevertheless important to notice that except for specific population where strong bottlenecks occurred, the relative risk provided by each risk allele is always less than 1.4, with *p*-values that are clearly significant due to the quite large number of patients analyzed in each of the studies, this number being even increased by gathering of samples in subsequent meta-analyses. The general question of ‘missing heritability’ [88] of complex phenotypes is the discrepancy visible between the calculated heritability, the ratio between the genetic part of the variance and the total phenotypic variance (around 50% in endometriosis) on the one hand and the part explained by the genes identified on the other hand (less than 10% in the current state of the art in the field). Multiple explanations have been proposed to explain the discrepancy. Above all, it is clear that the heterogeneity of a complex disease is a key problem, when similar phenotypes (but possibly different if analyzed in detail) are aggregated to perform genetic studies. Therefore, a better classification of the patients according to clinical characteristics is susceptible to improve the detection of genes that will strongly participate into the genetics of endometriosis but on a limited subset of patients. Another explanation is that deleterious variants are strongly counter-selected, leading therefore probably to low allelic frequencies for the mutant allele (Minor Allele Frequency below 1% for instance). These variants are generally not present in the microarray tools used for genotyping human samples, since the maximal polymorphism is searched for, constituting the array and bringing maximal information. Therefore, risk variants are generally absent from the arrays. Exome sequencing of families are on the contrary analyzed after performing a specific filtering against frequent variants (based upon the known frequencies in various populations emanating from the human genome projects). Another issue is that epistatic interactions are not specifically searched for by the various computational programs used to analyze GWAS, that are essentially based upon mono-locus analyses. In the future, with the significant and regular increase of computing power, such questions will probably be addressed, and allow to unravel groups of variants acting epistatically on the risk of developing the diseases.

Exome-sequencing approaches on the other hand are deliberately focused upon the genetic causes in a given family or a small number of families. The genes found by these approaches are unlikely to provide a complete description of the genetic landscape of endometriosis. Nevertheless, each gene found, through its network of interactant proteins will help completing the image. Besides, as mentioned before, there is a large part of endometriosis that is related to non-genetic parameters, either through stochastic reasons, or through environmental exposures. Nevertheless, until now no really convincing causal environmental factor has been found in endometriosis, as recently reviewed [102], albeit some scanty evidence suggest potential links with bisphenol A, phthalates and organochlorinated components [103].

## Figures and Tables

**Table 1:** List of Genome Wide Association Studies.

REFERENCE	PAPER PMID	ETHNICITY	STUDY TYPE	CASES (N)	CONTROLS (N)	ENDOME TRIOSIS DIAGNOSIS	CONTROLS SELECTION	STAGES OF THE STUDY	ASSOCIATED SNPs	SNP NAMES
**(a) Discovery Study**										
Uno et al., 2010 [30]	20601957	Asian	Case-control of hospital based	1907	5292	Laparoscopy	Laparoscopy	Discovery	1	rs10965235 (CDKN2BAS)
Adachi et al., 2010 [31]	20844546	Asian	Meta-analysis of population and hospital based	696	825	Medical history, MRI and laparoscopy	Medical history, MRI and laparoscopy	Discovery	5	rs6542095, rs11677416, rs3783550 and rs3783525 (near IL1 A gene) and rs801112 (RHOU)
Painter et al., 2011 [32]	3019124	Oceania and European	Meta-analysis of population and hospital based	3194	7060	Laparoscopy	Men, medical history and laparoscopy	Discovery	1	rs12700667 (NFE2L3-HOXA10)
Nyholt et al., 2012 [33]	23104006	Oceania and European	Meta-analysis of population and hospital based	4604	9393	Laparoscopy	Men, medical history and laparoscopy	Discovery	7	rs12700667 (NFE2L3-HOXA10 ), rs75211902 (WNT4), rs1537377 (CDKN2B-AS1), rs10859871 (VEZT), rs4141819 (ETAA1), rs77399264 (ID4), rs13394619 (GREB1)
Pagliardini et al., 2013 [34]	23142796	Asian and European	Meta-analysis of population and hospital based	305	2710	Laparoscopy	Laparoscopy blood donors	Discovery	3	rs1333049 (CDKN2BAS), rs7521902 (WNT4), rs1250248 (FN1)
Albertsen et al., 2013 [35]	23472165	European	Case-control of hospital base	2019	14,471	Laparoscopy	Laparoscopy	Discovery	3	rs2235529 (LINC00339-WNT4),rs1519761 and rs6757804 (RND3)
Rahmioglu et al., 2014 [36]	24676469	American, European and Oceania	Meta-analysis of population and hospital based	11,506	32,678	Laparoscopy	Laparoscopy	Discovery	6	rs12700667 (NFE2L3-HOXA10),rs7521902 (WNT4), rs10859871 (VEZT) rs1537377 (CDKN2B-AS1), rs7739264 (ID4), rs13394619 (GREB1)
Borghese et al., 2015 [37]	25722978	European	Meta-analysis of population and hospital based	60	20	Laparoscopy	Laparoscopy	Discovery	4	rs4703908 (ZNF366),rs227849 (RUNX2/ SUPT3H), rs2479037 (VTI1A) and rs966674 (NA)
Sapkota et al., 2015 [38]	26337243	Asian, African, European and Oceania	Meta-analysis of population and hospital-based	998	783	Laparoscopy	Laparoscopy	Discovery	6	rs7521902 (WNT4), rs13394619 in GREB1, rs12700667 (NFE2L3-HOXA10), rs6542095 (IL1A, rs7739264 (ID4) and rs1537377 (CDKN2B-AS1)
Wang et al., 2016 [39]	27506219	Asian	Case-control of hospital based	1448	1540	Laparoscopy	Laparoscopy	Discovery	3	rs11692361 (MEIS1), rs10256972 (C7orf50), rs4966038 (IGF-1R)
Uimari et al., 2017 [40]	28333195	Oceania and European	Case-control of hospital-based	3194	7060	Laparoscopy	Men, medical history and laparoscopy	Discovery	1	rs144240142 (MAP3K4)
Sapkota et al., 2017 [41]	28537267	Asian, Oceania European and American	Meta-analysis of population and hospital-based	17,045	191,596	Laparoscopy	Medical history and laparoscopy	Discovery	19	rs12037376 (WNT4), rs11674184 and rs77294520 (GREB1), rs6546324 ( ETAA1), rs4762326 (VEZT), rs10167914 (IL1A), rs1903068 (KDR), rs760794 (ID4),rs74485684 (FSHB), rs12700667 (NFE2L3-HOXA10), rs1537377, rs1075727 and rs1448792 (CDKN2B-AS1), rs1250241 (FN1), rs1971256 (CCDC170), rs71575922 and rs17803970 (SYNE1) rs2206949 (ESR1), rs74491657 (7p12.3)
Sobalska-kwapis et al., 2017 [42]	28881265	European	Case-control of hospital based	171	2934	Laparoscopy	Medical History	Discovery	19	18 SNPs (near of C2)and rs10129516 (PARP1P2-RHOJ)
Sapkota et al., 2017 [43]	28900119	Oceania, European and American	Meta-analysis of population and hospital based	9000	150,001	Medical History Laparoscopy	Medical History and Laparoscopy	Discovery	3	rs13394619 (GREB1 at 2p25.1), rs1801262 (CUBN), gene-level (CIITA et PARP4)
Painter et al., 2018 [44]	29608257	European	Case-control of hospital based	3194	2057	Laparoscopy	Laparoscopy	Discovery	13	rs2475335 (PTRD), rs9865110 (PDZRN3-CNTN3), rs2278868 (SKAP1), rs12303900 (KITLG-DUSP6) rs9349553(TFAP2D), rs10008492 (KLF3-TLR10), rs9530566 (LMO7-KCTD12), rs17693745 (CEP112), rs1755833 (PRIM2), rs7515106 (WNT4-ZBTB40), rs10459129 (PARP11-CCND2) rs2198894 (ZNF536-TSHZ3), rs7042500 (THEM215-APTX)
Christofolini et al., 2019 [45]	30044155	European	Case-control of hospital based	394	650	Laparoscopy	Laparoscopy	Discovery	2	rs10928050 (KAZN) and rs2427284 (LAM5)
Adewuyi et al., 2020 [46]	32121467	European, Asian and American	Meta-analysis of population and hospital based	76,728	507,936	Medical history and laparoscopy	Medical History	Discovery	3	SNPs nears ARL14EP , TRIM32, and SLC35G6
**(b) Replication Study**										
Painter et al., 2011 [32]	3019124	Oceanie	Meta-analysis of population and hospital based	2392	2271	Laparoscopy	Men, medical history and laparoscopy	Replication	1	rs12700667 (NFE2L3-HOXA10)
Nyholt et al., 2012 [33]	23104006	Asian Oceanie	Meta-analysis of population and hospital based	1044	4017	Laparoscopy	Men, medical history and laparoscopy	Replication	7	rs12700667 (NFE2L3-HOXA10 ), rs75211902 (WNT4), rs1537377 (CDKN2B-AS1), rs10859871 (VEZT),rs4141819 (ETAA1), rs77399264 (ID4), rs13394619 (GREB1)
Albertsen et al., 2013 [35]	23472165	European	Meta-analysis of population and hospital based	505	1811	Laparoscopy	Laparoscopy	Replication	3	rs2235529 (LINC00339-WNT4),rs1519761 and rs6757804 (RND3)
Sapkota et al., 2015 [38]	26337243	Asian, African	Meta-analysis of population and hospital based	998	783	Laparoscopy	Laparoscopy	Replication	6	rs7521902 (WNT4), rs13394619 in GREB1 rs12700667 (NFE2L3-HOXA10)
Osiński et al., 2018 [47]	30010178	European	Case-control of hospital base	315	406	Laparoscopy	Medical History	Replication	2	rs12700667 (NFE2L3-HOXA10) and rs4141819 (ETAA1)
**(c) Connection between Endometriosis and Others Pathologies**										
Painter et al., 2011 [32]	3019124	Oceanie and European	Meta-analysis of population and hospital based	3194	7060	Laparoscopy	Men, medical history and laparoscopy	Discovery	1	rs12700667 (NFE2L3-HOXA10)
Gallagher et al., 2019 [48]	31649266	European	Case-control of hospital base	35,474	267,505	Medical history, MRI	Medical history, MRI	Discovery	4	rs58415480 (ESR1), rs11031006 (FSHB), rs35417544 (GREB1), rs7412010 (WNT4)
Adewuyi et al., 2020 [46]	32959083	European, Asian and Americanian	Meta-analysis of population and hospital based	187,810	521,301	Laparoscopy	Medical History and laparoscopy	Discovery	26	rs116810322 (TRIM26), rs11793648 (TRIM32), rs9891297 (SLC35G6), rs1395455 (CSF3R), rs1620977 (NEGR1), rs12121863 (CRB1), rs9586 (KLHDC8B), rs9835157 (IP6K1), rs12512642 ( SCLT1), rs13164188 (NUDT12), rs7933594 (TYR), rs6680839 (TNR), rs72740410 (BRINP3), rs13118306 (CC2D2A), rs2134025 (TACR3) rs9347896 (C6orf1), rs11784932 (GSDMC), rs9538160 (PCDH17), rs35625885 (NR2F2), rs6808036 (CCDC36), rs323509 (NUDT12), rs6788293 (LINC00620)

GWA: Genome wide association study, MRI: magnetic resonance imaging, PMID: Pubmed identifer, RS: RefSNP, SNP: single nucleotide polymorphism.

**Table 2:** Review of significant SNPs in the different studies.

Locus	Gene(s) of Interest	SNP	Cases (N)	Controls (N)	RA	OA	Relative Risk	*p*-Value	Paper PMID
1p31.1	NEGR1	rs1620977	187,810	521,301	A	G	1.027	10^−8^ (10^−2^) *	32959083
1p34.3	CSF3R	rs1395455	187,810	521,301	A	G	1.025	10^−8^ (10^−2^) *	32959083
1p36.12	WNT4	rs7521902	305	2710	A	C	1.2	10^−9^	23142796
		rs7521902	11,506	32,678	A	C	1.18	10^−15^	24676469
		rs4654783	2019	14,471	A	G	1.21	10^−9^	23472165 **
		rs2235529	2019	14,471	A	G	1.29	10^−9^	23472165 **
		rs7521902	4604	9393	A	C	1.18	10^−8^	23104006
		rs7521902	1044	4017	A	C	NA	10^−5^	23104006 **
		rs7521902	998	783	A	C	1.18	10^−8^	26337243
		rs7521902	998	783	A	C	1.3	10^−3^	26337243 **
		rs7521902	23,671	68,894	A	C	1.29	10^−15^	27470151
		rs7739264	998	783	T	C	1.14	10^−7^	26337243
		rs7515106	3194	2057	C	T	1.09	10^−5^	29608257 **
		rs12037376	17,045	191,596	A	G	1.16	10^−17^	28537267
		rs7412010	35,474	267,505	C	G	1.13	10^−29^	31649266
1p36.21	KAZN	rs10928050	394	650	A	G	1.31	10^−2^	30044155 **
1q25.1	TNR	rs6680839	187,810	521,301	T	C	0.975	10^−10^ (10^−4^) *	32959083
1q31.1	BRINP3	rs72740410	187,810	521,301	T	C	1.048	10^−8^ (10^−2^) *	32959083
1q31.3	CRB1	rs12121863	187,810	521,301	A	T	0.971	10^−8^ (10^−2^) *	32959083
1q42.13	RHOU	rs801112	696	825	A	T	1.65	10^−6^	20844546
2p14	ETAA1	rs6546324	998	191,596	A	C	1.08	10^−9^	28537267
		rs4141819	4604	9393	C	T	1.15	10^−8^	23104006
		rs4141819	1044	4017	C	T	NA	10^−2^	23104006 **
		rs4141819	11,506	32,678	C	T	1.08	10^−6^	24676469
		rs4141819	315	406	C	T	1.35	10^−2^	30010178 **
2p14	MEIS1	rs11692361	1448	1540	C	T	0.70	10^−7^	27506219
2p25.1	GREB1	rs13394619	11,506	32,678	G	A	1.13	10^−8^	24676469
		rs13394619	998	783	G	A	1.15	10^−9^	26337243
		rs13394619	998	783	G	A	1.13	10^−2^	26337243 **
		rs13394619	7164	21,005	A	G	0.89	10^−9^	28900119
		rs13394619	4604	9393	A	G	1.15	10^−8^	23104006
		rs13394619	1044	4017	A	G	NA	10^−3^	23104006 **
		rs11674184	17,045	191,596	T	G	1.13	10^−17^	28537267
		rs77294520	17,045	191,596	C	G	1.16	10^−13^	28537267
		rs35417544	35,474	267,505	T	C	1.09	10^−13^	31649266
2q13	IL1A	rs6542095	696	825	C	T	1.5	10^−6^	20844546
		rs6542095	998	783	C	T	1.22	10^−10^	26337243 **
		rs6542095	998	783	C	T	1.26	10^−2^	26337243
		rs6542095	7164	21,005	T	C	0.90	10^−7^	28900119
		rs11677416	696	825	T	A	2.0	10^−6^	20844546
		rs10167914	17,045	191,596	G	A	1.15	10^−9^	28537267
		rs3783550	696	825	C	A	1.51	10^−6^	20844546
		rs3783525	696	825	A	T	1.52	10^−6^	20844546
2q23.3	RND3	rs1519761	2019	14,471	C	A	1.20	10^−7^	23472165 **
		rs6734792	2019	14,471	G	A	1.20	10^−8^	23472165 **
		rs1519761	2019	14,471	G	A	1.20	10^−8^	23472165 **
		rs6757804	2019	14,471	G	A	1.20	10^−8^	23472165 **
		rs6734792	11,506	32,678	C	T	1.10	10^−6^	24676469
2q35	FN1	rs1250241	17,045	191,596	T	A	1.23	10^−9^	28537267
		rs1250248	305	2710	A	G	1.13	10^−9^	23142796
		rs1250248	1129	831	A	G	1.17	10^−2^	23315067 **
		rs1250248	11,506	32,678	A	G	1.11	10^−4^	24676469
3p13-3p12.3	PDZRN3-CNTN3	rs9865110	3194	2057	C	A	1.1	10^−6^	29608257
3p21.31	KLHDC8B	rs9586	187,810	521,301	T	C	0.976	10^−8^ (10^−4^) *	32959083
3p21.31	IP6K1	rs9835157	187,810	521,301	A	G	1.034	10^−8^ (10^−4^) *	32959083
3p21.31	CCDC36	rs6808036	187,810	521,301	T	G	1.044	10^−8^ (10^−5^) *	32959083
3p25.1	LINC00620	rs6788293	187,810	521,301	T	C	0.952	10^−8^ (10^−2^) *	32959083
4p14	KLF3-TLR10	rs10008492	3194	2057	T	C	1.14	10^−5^	29608257
4p15.32	CC2D2A	rs13118306	187,810	521,301	C	G	0.977	10^−8^ (10^−3^) *	32959083
4q12	KDR	rs1903068	17,045	191,596	A	G	1.11	10^−15^	28537267
4q24	TACR3	rs2134025	187,810	521,301	A	G	1.029	10^−8^ (10^−4^) *	32959083
4q28.2	SCLT1	rs12512642	187,810	521,301	T	C	1.28	10^−8^ (10^−2^) *	32959083
5	NA	rs966674	288	259	C	G	2.95	10^−3^	25722978
5q13.1	ZNF366	rs4703908	288	259	C	G	2.22	10^−3^	25722978
5q21.2	NUDT12	rs13164188	187,810	521,301	T	C	1.025	10^−9^ (10^−2^) *	32959083
5q21.2	NUDT12	rs323509	187,810	521,301	A	C	1.042	10^−8^ (10^−2^) *	32959083
6p11.2	PRIM2	rs1755833	3194	2057	A	G	0.93	10^−5^	29608257
6p12.3	TFAP2D	rs9349553	3194	2057	T	C	1.09	10^−6^	29608257
6p21.1	RUNX2/SUPT3H	rs227849	288	259	A	G	2.31	10^−3^	25722978
6p21.33	C2	rs644045	171	2934	T	C	1.90	10^−10^	28881265
6p22.1	TRIM26	rs116810322	187,810	521,301	T	C	1.042	10^−9^ (10^−5^)	32959083
6p22.3	ID4	rs7739264	11,506	32,678	T	C	1.11	10^−10^	24676469
		rs7739264	4604	9393	T	C	1.17	10^−7^	23104006
		rs7739264	1044	4017	T	C	NA	10^−4^	23104006 **
		rs7739264	998	783	T	C	1.14	10^−7^	26337243
		rs7739264	23,671	68,894	T	C	1.10	10^−10^	27470151
		rs760794	17,045	191,596	T	C	1.17	10^−7^	28537267
		rs6907340	2019	14,471	A	G	1.12	10^−7^	23472165
6q12	ESY	rs12206488	187,810	521,301	A	G	0.976	10^−9^ (10^−3^) *	32959083
6q24.2	HIVEP2	rs2328370	187,810	521,301	A	C	1.023	10^−9^ (10^−3^) *	32959083
6q25.1	CCDC170	rs1971256	17,045	191,596	C	T	1.09	10^−8^	28537267
6q25.1	SYNE1	rs71575922	17,045	191,596	G	C	1.11	10^−8^	28537267
		rs17803970	17,045	191,596	A	T	1.15	10^−8^	28537267
6q25.1-q25.2	ESR1	rs2206949	17,045	191,596	T	C	1.10	10^−7^	28537267
		rs58415480	35,474	267,505	C	G	1.19	10^−7^	31649266
6q26	MAP3K4	rs144240142	3194	7060	T	C	1.71	10^−8^	28333195
6q27	C6orf1	rs9347896	187,810	521,301	A	G	1.29	10^−8^ (10^−2^) *	32959083
7p15.2	NFE2L3/HOXA10	rs12700667	3194	7060	A	G	1.22	10^−7^	3019124
		rs12700667	2392	2271	A	G	1.17	10^−3^	3019124 **
		rs12700667	4604	9393	A	G	1.18	10^−10^	23104006
		rs12700667	1044	4017	A	G	NA	10^−9^	23104006 **
		rs12700667	998	783	A	G	1.19	10^−10^	26337243
		rs12700667	11,506	32,678	A	G	1.13	10^−9^	24676469
		rs12700667	17,045	191,596	A	G	1.1	10^−10^	28537267
		rs12700667	315	406	A	G	1.3	10^−2^	30010178 **
7p22.3	C7orf50	rs10256972	1448	1540	A	C	1.30	10^−6^	27506219
7q31.1	DNAJB9	rs11561993	187,810	521,301	T	C	1.025	10^−9^ (10^−3^) *	32959083
8q24.21	GSDMC	rs11784932	187,810	521,301	A	C	1.026	10^−8^ (10^−4^) *	32959083
9p21.1	THEM215-APTX	rs7042500	3194	2057	A	G	0.9	10^−5^	29608257
9p21.1	ACO1	rs13299293	187,810	521,301	A	T	0.976	10^−9^ (10^−3^) *	32959083
9p21.3	CDKN2B-AS1	rs10965235	1907	5292	C	A	1.44	10^−12^	20601957
		rs1537377	11,506	32,678	C	T	1.12	10^−8^	24676469
		rs1537377	17,045	191,596	T	C	1.21	10^−9^	28537267
		rs1537377	4604	9393	C	T	1.15	10^−6^	23104006
		rs1537377	1044	4017	C	T	NA	10^−4^	23104006 **
		rs1448792	17,045	191,596	G	A	1.08	10^−8^	28537267
		rs10757272	17,045	191,596	C	T	1.07	10^−7^	28537267
9p24.1-p23	PTRD	rs2475335	3194	2057	T	C	1.11	10^−8^	29608257
9p24.1-p23	PTPRD	rs1931391	187,810	521,301	T	G	1.031	10^−8^ (10^−2^) *	32959083
9q33.1	TRIM32	rs11793648	46,262	364,789	NA	NA	NA	10^−6^	32121467
10p13	CUBN	rs1801232	7164	21,005	T	G	0.86	10^−7^	28900119
10q11.21	HNRNPA3P1	rs10508881	2019	14,471	A	G	1.19	10^−7^	23472165 **
10q11.21	LOC100130539								
10q25.2	VTI1A	rs2479037	288	259	C	T	4.36	10^−3^	25722978
11p14.1	FSHB	rs74485684	17,045	191,596	G	A	1.11	10^−8^	28537267
		rs11031006	35,474	267,505	A	G	1,10	10^−15^	31649266
11p14.1	ARL14EP	rs4071559	46,262	364,789	NA	NA	NA	10^−7^	32121467
11q14.3	TYR	rs7933594	187,810	521,301	C	G	1.024	10^−9^ (10^−2^) *	32959083
12p13.32	PARP11-CCND2	rs10459129	3194	2057	A	G	0.9	10^−5^	29608257
12q21.32-12q21.33	KITLG-DUSP6	rs12303900	3194	2057	G	T	1.28	10^−7^	29608257
12q22	VEZT	rs10859871	11,506	32,678	C	A	1.18	10^−15^	24676469
		rs10859871	4604	9393	C	A	1.20	10^−9^	23104006
		rs10859871	23,671	68,894	C	A	1.19	10^−20^	27470151
		rs10859871	998	783	C	A	1.16	10^−7^	26337243
		rs10859871	1044	4017	C	T	NA	10^−6^	23104006 **
		rs4762326	17,045	191,596	T	C	1.08	10^−8^	28537267
13q21.1	PCDH17	rs9538160	187,810	521,301	A	G	0.976	10^−8^ (10^−3^) *	32959083
13q22.2-13q22.3	LMO7-KCTD12	rs9530566	3194	2057	C	A	1.08	10^−5^	29608257
14q23.2-14q23.2	PARP1P2/RHOJ	rs10129516	171	2934	T	C	3.01	10^−10^	28881265
15q26.2	NR2F2	rs35625885	187,810	521,301	A	G	0.965	10^−8^ (10^−2^) *	32959083
15q26.3	IGF-1R	rs4966038	1448	1540	C	G	1.40	10^−9^	27506219
17p13.1	SLC35G6	rs9891297	46,262	364,789	NA	NA	NA	10^−6^	32121467
17q21.32	SKAP1	rs2278868	3194	2057	C	T	0.92	10^−6^	29608257
17q24.1	CEP112	rs17693745	3194	2057	T	C	1.08	10^−5^	29608257
19q12	ZNF536-TSHZ3	rs2198894	3194	2057	T	C	1.09	10^−5^	29608257
20q13.33	LAMA5	rs2427284	394	650	A	G	0.49	10^−3^	30044155 **

OA: Other allele, RA: Risk allele, PMID: Pubmed identifer, RS: refSNP, SNP: Single nucleotide polymorphism. * *p* value meta-analysis for depression and endometriosis study (*p* value for endometriosis), ** Results from replication studies.

## Data Availability

Not applicable.
